# Costs of facility-based HIV testing in Malawi, Zambia and Zimbabwe

**DOI:** 10.1371/journal.pone.0185740

**Published:** 2017-10-16

**Authors:** Lawrence Mwenge, Linda Sande, Collin Mangenah, Nurilign Ahmed, Sarah Kanema, Marc d’Elbée, Euphemia Sibanda, Thokozani Kalua, Gertrude Ncube, Cheryl C. Johnson, Karin Hatzold, Frances M. Cowan, Elizabeth L. Corbett, Helen Ayles, Hendramoorthy Maheswaran, Fern Terris-Prestholt

**Affiliations:** 1 Zambart, Lusaka, Zambia; 2 Malawi-Liverpool-Wellcome Trust Clinical Research Programme, Blantyre, Malawi; 3 Centre for Sexual Health and HIV AIDS Research, Harare, Zimbabwe; 4 Faculty of Public Health and Policy, London School of Hygiene and Tropical Medicine, London, United Kingdom; 5 Department of HIV and AIDS, Ministry of Health, Lilongwe, Malawi; 6 Ministry of Health and Child Care, Harare, Zimbabwe; 7 Department of HIV/AIDS, World Health Organization, Geneva, Switzerland; 8 Population Services International, Harare, Zimbabwe; 9 Liverpool School of Tropical Medicine, Liverpool, United Kingdom; 10 Faculty of Infectious and Tropical Diseases, London School of Hygiene and Tropical Medicine, London, United Kingdom; 11 Division of Health Sciences, University of Warwick Medical School, Coventry, United Kingdom; Boston University, UNITED STATES

## Abstract

**Background:**

Providing HIV testing at health facilities remains the most common approach to ensuring access to HIV treatment and prevention services for the millions of undiagnosed HIV-infected individuals in sub-Saharan Africa. We sought to explore the costs of providing these services across three southern African countries with high HIV burden.

**Methods:**

Primary costing studies were undertaken in 54 health facilities providing HIV testing services (HTS) in Malawi, Zambia and Zimbabwe. Routinely collected monitoring and evaluation data for the health facilities were extracted to estimate the costs per individual tested and costs per HIV-positive individual identified. Costs are presented in 2016 US dollars. Sensitivity analysis explored key drivers of costs.

**Results:**

Health facilities were testing on average 2290 individuals annually, albeit with wide variations. The mean cost per individual tested was US$5.03.9 in Malawi, US$4.24 in Zambia and US$8.79 in Zimbabwe. The mean cost per HIV-positive individual identified was US$79.58, US$73.63 and US$178.92 in Malawi, Zambia and Zimbabwe respectively. Both cost estimates were sensitive to scale of testing, facility staffing levels and the costs of HIV test kits.

**Conclusions:**

Health facility based HIV testing remains an essential service to meet HIV universal access goals. The low costs and potential for economies of scale suggests an opportunity for further scale-up. However low uptake in many settings suggests that demand creation or alternative testing models may be needed to achieve economies of scale and reach populations less willing to attend facility based services.

## Introduction

Over 35 million people are living with HIV, the majority in sub-Saharan Africa [1]. In particular, HIV prevalence stands at 10.6%, 12.3% and 14.6% among individuals aged 15–64 in Malawi, 15–59 in Zambia and 15–64 in Zimbabwe, respectively [2–5]. Timely initiation of antiretroviral treatment (ART) has the potential to ensure those infected can lead healthy lives, potentially living as long as uninfected individuals in the region [6], and reduces the probability for further sexual and vertical transmission through suppressed viral load [3, 7]. Despite efforts to increase access to ART in the region, millions continue to die [1], while those who do start treatment do so late [8]. Achieving universal and timely access to ART relies on ensuring those who are infected with the virus are aware of their status [9].

In the last decade Southern Africa has seen significant scale up of HIV testing services (HTS). In Zambia, this has led to the proportion of 15-49-year-olds who have tested and received their HIV test result in the previous 12 months increasing from 19% of women and 12% of men in 2007 to 70% of women and 63% of men in 2015 [3]. According to the Malawi Population-Based HIV Impact Assessment (MPHIA), 76% of women and 67% of men aged 15–64 who are living with HIV know their HIV status [10]. In Zimbabwe, 71% of women and 70% of men aged between 15 and 64 who are living with HIV know their HIV status [4]. Conversely, though national statistics group all HTS indicators together, it is known that the scope of HTS has expanded beyond facility based activities [11]. For example community based HTS has been said to increase number of individuals with known HIV status and improve HIV knowledge in general [12–15]. This has mainly been achieved by increasing the availability of health facility-based HTS [16, 17].

Moreover countries have adopted the 2015 World Health Organisation (WHO) guidelines, which recommend immediate ART for all HIV-positive adults and children [18], and are aiming to achieve the UNAIDS 90-90-90 target (i.e. by 2020 90% of all people living with HIV should know their HIV status, 90% of all individuals with diagnosed HIV infection will receive sustained ART, and 90% of all individuals receiving ART will have viral suppression [19]. Clearly meeting these goals requires further scale-up and better targeting of HTS. Understanding the costs of delivering HTS is critical to ensure efficient use of resources and improve planning and budgeting. However, information on HTS costs remains sparse in the region, and where available, estimates show wide variation in costs per person tested ranging from US$5 to US$50 [20, 21].

This paper presents the costs of health provider delivered facility-based HTS in Malawi, Zambia and Zimbabwe and explores cost drivers and economies of scale. In addition, cost estimates presented in this paper will inform the cost-effectiveness analysis of HIVST implementation in the HIV-Self Testing AfRica (STAR) project.

## Methods

### Setting

In 2016 UNITAID commissioned STAR project to assess the feasibility, acceptability and the potential health impact of distributing HIV self-test kits in Malawi, Zambia and Zimbabwe. We undertook a cost analysis of facility-based HTS services provided at 54 health facilities serving the STAR study populations in Malawi (15), Zambia (10) and Zimbabwe (29). Health facilities included both primary and secondary care facilities.

In the STAR project community-based distribution of HIVST is being evaluated in Malawi, Zambia and Zimbabwe. In these countries, communities were selected for the purposes of the main implementation evaluation being undertaken. Briefly, communities were selected in collaboration with the countries’ Ministry of Health. The selected communities had to be served by a local government health facility providing HIV care, with no alternative HIV care facility nearby. Preference was given to communities with high HIV prevalence. For this costing study, in Malawi and Zimbabwe all health facilities included in the impact evaluation were included while in Zambia 12 facilities were randomly selected. Data collection occurred prior to HIVST implementation.

In Malawi, all 15 facilities were rural primary health clinics located in Blantyre, Machinga, Mwanza and Neno districts. In Zambia, there were two peri-urban and eight rural primary health clinics located in four districts, Ndola, Kapiri Mposhi, Choma and Lusaka. In Zimbabwe, all 29 health facilities evaluated were in rural areas including one mission hospital, one mine hospital, two district government hospitals, and 25 rural primary health clinics. There were between one and six HIV testing staff full-time equivalents (FTEs) working at each health facility in the three countries. For Zambia, unlike Malawi and Zimbabwe, HIV testing staff included a mix of paid and volunteer counselors. Table 1 presents a detailed description of study sites.

**Table 1:** Sample overview and facility description.

Characteristic	Description	Malawi	Zambia	Zimbabwe
Number of districts	Number of districts	4	3	6
Number of sites	Sample size	15	10	29
Type of facility	Primary health clinic (Hospital)	15 (0)	10 (0)	27 (3)
Population	Mean catchment population at sampled facilities (median; range^$^)	27,439 (19,172; 5,500–82,581)	18,266 (15,223; 7673–50,094)	3,196 (3,088; 549–6,699)
Location	Rural (urban/peri-urban)	15 (0)	8 (2)	29 (0)
Personnel	Mean HTS* FTEs^&^ per facility (median; range^$^)	2 (2; 1–4)	6 (6; 2–10)	5 (4; 2–11)
	Mean HTS FTEs per 10,000 population (median, Range)	16 (13; 5–35)	31 (31; 13–53)	68 (52; 24–184)
	Mean Paid counsellors per facility (median; range^$^)	2 (2; 1–4)	1 (1; 0–5)	5 (4; 2–11)
	Mean Volunteers per facility (median; range^$^)	-	4 (4; 2–7)	-
National HIV prevalence (%)[2–5]	Adults 15 to 49 years	9.1	12.3	14.6

FTE = Full time Equivalent.

^&^HTS = HIV testing services.

^$^Range is presented in terms of minimum—maximum.

At all health facilities individuals may voluntarily attend the health facility to request HIV testing or may be referred to the HTS service because they are unwell, pregnant or have an illness that warrants HIV testing (e.g. Tuberculosis). In all three countries, HIV testing is performed using finger-prick rapid diagnostic test (RDT) kits and follows standard serial testing algorithms where those who test positive on the first RDT undergo confirmatory testing using a different RDT kit [22]. In each of the countries, a different RDT kit is used for the confirmatory testing. For those found to have discordant test results on serial testing are an immediate parallel repeat test is done on both testing is done on both tests. For those found to have discordant test results on serial testing an immediate parallel repeat test is done. If both test 1 and test 2 are reactive results are reported positive; if both are non-reactive, results are reported negative. If the results from parallel testing are discordant, clients are advised to repeat HIV test after 4 weeks in Malawi and 14 days in Zambia and Zimbabwe. All those who test HIV-negative are advised to re-test in three months. A detailed description of the national HIV testing algorithms in the three countries is provided in the Supplemental figures S1–S3 Figs. HTS department is a unit in the facility with a physical space where all HTS data within the facility are aggregated. HIV testing is done by trained counselors, either employed or volunteers, at the facility. Counselors may also be placed in different locations within the facility (e.g. Antenatal clinic) to perform HIV testing.

### Cost data collection

The study was undertaken from the health providers’ perspective to estimate the costs of routine provider delivered facility-based HTS and understand key determinants of these costs. Full annual financial and economic costs were estimated. Financial costs represent all expenditures for resources used in the intervention, while economic costs capture the full value of all resources used, including valuation of donated goods or services, here the opportunity cost of volunteer counsellors’ time [23]. Volunteer time was valued as a product of the number of hours that volunteers spent on doing HTS activities and the average stipend rate which non-government organizations (NGOs) pay volunteers for providing similar activities in Zambia. Annual resource use data were sequentially and retrospectively collected with end dates rolling between June 2016 and April 2017, depending on the date of the data collection visit. Costs were adjusted to 2016 United States dollars (US$) using the average exchange rates, ZMK722.99 for Malawi, ZMW10.03 for Zambia and US$1 for Zimbabwe, over the period of the costing [24] and deflators [25].

Standardised costing methods were developed collaboratively by economists across the three countries to ensure consistency of data collection and analysis. We employed both ingredients based (bottom up) costing and top-down costing where we apportioned costs stepwise to their respective cost centers [10, 26]. Types, quantities and unit costs of cost items were collected through interviews, expenditure and outcome review at facility and district levels. Where unit costs were not present in the expenditure records, market prices were used. See S1 Table for details of the allocation of each cost item. Capital costs included: buildings, equipment and vehicles whilst recurrent costs captured personnel, HIV testing commodities, general supplies, facility level operations including transportation and waste management. Capital costs were annualised and discounted at a 3% rate in accordance with WHO guidelines [27]. Overhead costs were considered at two levels; facility overhead which included all the costs that are needed to ensure the overall running of the facility, and HTS centre-specific costs, which are the costs of running the HTS department where HIV-related activities are conducted. Due to difference in financial reporting system across the three countries overhead costs were allocated differently in each country, particularly costs related to health systems management (Above-facility administration, supervision & mentorship) and facility administration. Supply chain costs were apportioned using allocation factors from literature [28]. See supplemental table S1 Table for details.

### Outputs and allocation factor data collection

Alongside cost data collection we collected data on the catchment population, number of outpatient department (OPD) visits, number of staff, number of HTS visits and number of HIV-positive results, through reviewing facility registers. Data sources were facility registers and heath information aggregation forms. These data were also used in the allocation of overhead and shared costs.

### Data analysis

Total annual costs of running HTS at each facility and the respective unit costs were estimated by dividing the total facility costs by the annual number of people tested and the number of HIV-positive individuals identified. Descriptive statistical analysis was performed to calculate mean and median (with the minimum and maximum ranges) for unit costs per HIV test and HIV-positive identified for each country. To explore potential drivers of costs descriptively, Pearson correlations were calculated. A univariate sensitivity analysis was undertaken to understand the impact of HIV test kit price and staff time on the unit costs. The impact of price on unit costs was explored by applying the lowest and highest observed test kit prices across the three countries. The impact of staffing was explored by considering variation in staffing in a +/-20% range to; (a) cope with increased testing demand; (b) explore impact of introduction of community-based HIV testing or HIV self-testing requiring fewer facility based counsellors. We also assessed the impact of the size of facility on the unit costs in Zimbabwe, where the costing sample included both clinics and hospitals. All facilities from Malawi and Zambia were clinics; we only had a clinic-hospital mix in Zimbabwe (3 rural hospitals out of 29 facilities). In our analysis facility size is defined by the catchment population and HTS department by the number of annual HTS visits.

### Ethics

Ethical approvals for the project were secured from the appropriate research review boards. This included the London School of Hygiene and Tropical Medicine (LSHTM) Ethics Committee, Malawi National Health Sciences Research Committee, University of Zambia Biomedical Research Ethics Committee, Medical Research Council of Zimbabwe (MRCZ) and University College London Ethics Committee. The STAR trials are registered under the Clinical Trials Network (ClinicalTrials.gov) under registration numbers NCT02793804; NCT02718274; Pan African clinical trials registry (Zimbabwe) PACTR201607001701788.

## Results

### HTS output summary

The mean number of HIV tests conducted per clinic during the 12-month costing period was 2,359 with 3,404, 3,161 and 1,542 in Malawi, Zambia and Zimbabwe, respectively (Table 2). The mean HIV prevalence amongst those who accessed HIV testing at the health facilities was 7% (9% for Malawi, 9% for Zambia, and 6% for Zimbabwe). While the annual number of HTS visits was significantly associated with the size of the health facility catchment population when pooling across the three countries (R^2^ = 0.53, N = 53, P<0.000), when estimated at the country level the correlation only remained significant in Malawi ((R^2^ = 0.55, N = 15, P<0.002) and Zambia (R^2^ = 0.76, N = 10, P = 0.001) but no longer in Zimbabwe (R^2^ = 0.030, N = 28, P<0.379).

**Table 2:** Test kit prices and average (mean; median) annual facility HTS outputs.

	Malawi	Zambia	Zimbabwe
Test kit price^&^ First	Determine $1.00 Unigold $1.00	Determine $1.00-$1.20 Unigold $1.60	Determine $1.07 First response $0.71
HIV tests	3404 (3461; 835–7953)	2789 (2338; 852–6957)	1542 (1132; 368–5735)
HIV+ identified (median; range*)	304 (230;25–950)	251; (120; 48–907)	93; (63; 12–409)
Facility HIV+ reactivity rate	9% (8%; 3%-16%)	9% (7%; 2%-16%)	6% (6%; (1%-14%)

*Range is presented in terms of minimum—maximum

^&^Test kit prices were derived from national laboratory and medical supplies procurement catalogues from each country complimented by discussion with key stakeholder

**Fig 1:**
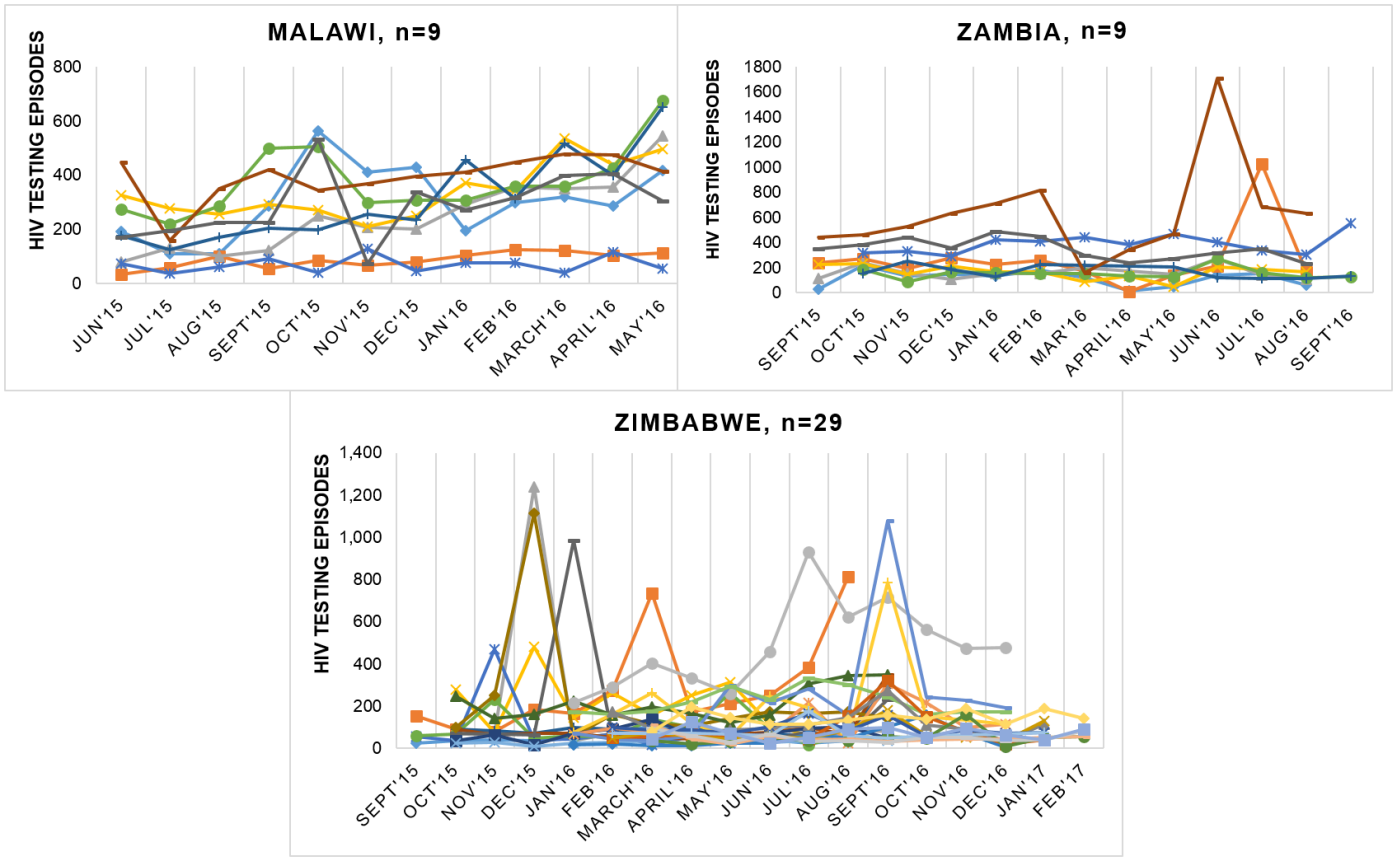
Monthly HTS visits by facility*. *monthly service statistics were not available for all clinics.

Fig 1 shows the number of HTS visits each month for all the health facilities sampled in the three countries. In Malawi, the majority of the health facilities appears to have experienced gradual increases in number of HTS visits over the study period. In Zambia, the number of HTS visits every month appears relatively constant over the year, with two clinics experiencing a peak in visits in July and August. In Zimbabwe, many of the health facilities experienced significant fluctuation in monthly HTS visits.

The mean annual number of HIV testing episodes per HTS staff FTE was 1132 (519–2075) in Malawi, 597 (238–1257) in Zambia and 895 (237–2285) in Zimbabwe. Country-level analysis did show the number of HTS staff was strongly correlated with the size of the facility catchment population in Zambia, though not in Malawi and Zimbabwe. Cross-country analysis shows that there was no significant relationship between the number of HIV counsellors employed at each health facility and the facility catchment population (R^2^ = 0.01, N = 53, P = 0.4039). At country-level, the results showed that the correlation was significant in Zambia, but not in Malawi and Zimbabwe. Overall, there was no correlation between the number of HIV counsellors employed and the number of HIV testing episodes (R^2^ = 0.01, N = 54, P = 0.53).

### Total HTS costs

**Fig 2:**
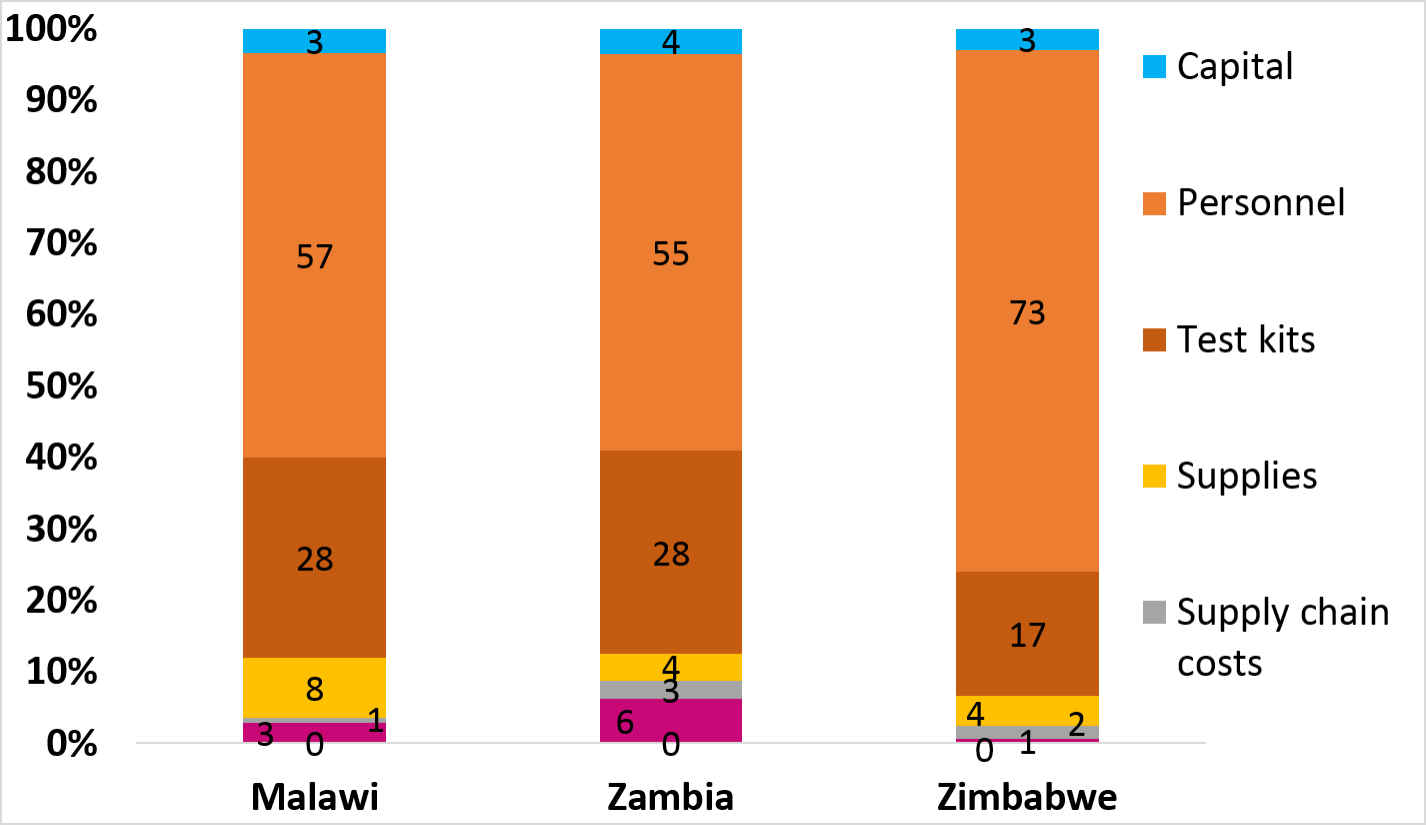
Input shares by country (%).

S2 Table presents resource utilization for key recurrent supplies. The total annual economic costs for the health facilities sampled in the three countries are shown in Table 3, financial costs are presented in Supplemental Table S3 Table. The median total annual costs were US$14,822 (range: US$5,386-US$25,124) for Malawi, US$8,797 (range: US$4,486-US$43,106) for Zambia and US$8,774 (range: US$4,476-US$38,514) for Zimbabwe. In the three countries, salaries for personnel accounted for 57%, 55% and 73% of the total annual cost in Malawi, Zambia and Zimbabwe, respectively (Fig 2). The variation in costs across the countries was significantly correlated with variation in staffing levels (P = 0.04 for Malawi, P = 0.04 for Zambia, and P<0.01 for Zimbabwe); some facilities relied heavily on volunteer/ lay providers (mainly in Zambia) whereas others tended to employ highly trained and paid staff. The cost of the HIV RDT kit and supplies accounted for 28% in Malawi, 28% in Zambia and 17% in Zimbabwe of the total annual cost. Capital costs accounted for approximately 4% of the total annual cost for Zambia, and 3% for Malawi and Zimbabwe.

**Table 3:** Total and mean economic costs (minimum-maximum).

Cost item	Malawi (US$)			Zambia (US$)			Zimbabwe (US$)		
	Total annual costs	Cost per test performed	Cost per HIV+	Total annual costs	Cost per test performed	Cost per HIV+	Total annual costs	Cost per test performed	Cost per HIV+
**Capital costs**									
*Buildings and storage*	347 (54–777)	0.13 (0.01–0.28)	1.9 (0.34–7.52)	133 (59–254)	0.07 (0.02–0.21)	0.97 (0.17–1.87)	190 (32–514)	0.22 (0.01–1.40)	4.62 (0.44–24.47)
*Equipment*	169 (57–300)	0.08 (0.01–0.28)	1.43 (0.12–8.70)	160 (41–391)	0.1 (0.01–0.46)	1.44 (0.06–3.35)	108 (38–304)	0.11 (0.01–0.45)	2.36 (0.15–11.16)
Vehicles	-	-	-	91 (21–249)	0.06 (0.01–0.26)	0.69 (0.04–1.86)	22 (0–633)	0.01 (0.00–18)	0.06 (0.00–1.77)
Other	-	-	-	43 (29–61)	0.02 (0.01–0.05)	0.39 (0.04–1.24)	-	-	-
*Total capital cost*	*517* *(162–938)*	*0*.*2* *(0*.*04–0*.*51)*	*3*.*33* *(0*.*61–16*.*22)*	*428* *(211–844)*	*0*.*24* *(0*.*05–1*.*00)*	*3*.*49* *(0*.*32–697)*	*320* *(72–1*,*095)*	*0*.*33* *(0*.*03–1*.*85)*	*7*.*04* *(0*.*66–32*.*36)*
**Recurrent costs**		-	-						
Personnel	8,375 (2,893–13,828)	2.97 (1.35–6.00)	46.57 (13.05–115.72)	6,678 (1,373–32,665)	2.05 (0.51–4.70)	36.83 (5.82–115.76)	7,670 (3,141–34,398)	6.69 (1.85–118.88)	131 (26.36–313)
Supplies—test kits	3,713 (912–9,064)	1.19 (1.13–1.26)	19.16 (8.51–41.58)	3421 (1,128–8,692)	1.22 (1.14–1.35)	21.34 (8.21–46.39)	1826 (439–6,747)	1.2 (1.12–1.29)	28.71 (9.39–84.61)
Supplies	1,231 (783–1,632)	0.46 (0.79–1.09)	7.83 (1.22–31.32)	450 (163–596)	0.21 (0.08–0.58)	3.32 (0.62–5.95)	441 (130–2,032)	0.38 (0.09–2.9)	7.82 (1.61–31.27)
Supply chain	111 (70–147)	0.04 (0.01–0.10)	0.7 (0.11–2.82)	307 (101–779)	0.11 (0.10–00.12)	1.91 (0.76–4.16)	203 (63–676)	0.14 (0.03–0.34)	3.22 (0.41–9.26)
Operation & maintenance	393 (67–1325)	0.36 (0.06–1.22)	3.64 (0.62–12.27)	751 (210–1,427)	0.42 (0.05–1.14)	6.85 (0.32–13.71	56 (0.00–682)	0.1 (0.00–01.15)	0.7 (0.00–8.42)
Recurrent training	-	-	-	-	-	-	-	-	-
Waste management	31 (2–136)	0.01 (0.00–0.05)	0.24 (0.01–1.46)	2 (1–4)	-	0.02 (0.00–0.07)	2.01 (0.38–7.32)	-	0.06 (0.00–0.43)
*Total recurrent costs*	*14304* *(4*,*4981–24228)*	*4*.*85* *(2*.*96–890)*	*76*.*24* *(25*.*50–199*.*22)*	*11*,*609* *(4*,*440–43*,*071)*	*4* *(2*.*34–6*.*19)*	*70* *(16*.*30–184*.*39)*	*10198* *(4198–4162)*	*8*.*46* *(3*.*33–20*.*68)*	*171*.*88* *(41-97-426*.*05)*
**Total cost / unit cost**	**14,822** **(5,386–25,124)**	**4.92** **(2.95–8.33)**	**79.58** **(26.45–215.44)**	**11,652** **(4,486–43,106)**	**4.24** **(2.49–6.24)**	**73.63** **(16.62–191.35)**	**10,517** **(4,476–38,514)**	**8.79** **(3.38–21.51)**	**178.92** **(43.81–442.43)**

### Unit costs

The median costs per individual tested for HIV in Malawi, Zambia and Zimbabwe were US$4.56, US$3.96, US$6.25, respectively. The median cost per HIV-positive individual identified were US$58.044 for Malawi, US$54.33 for Zambia and US$141.67 for Zimbabwe. Average unit costs are reported in Table 3.

**Fig 3:**
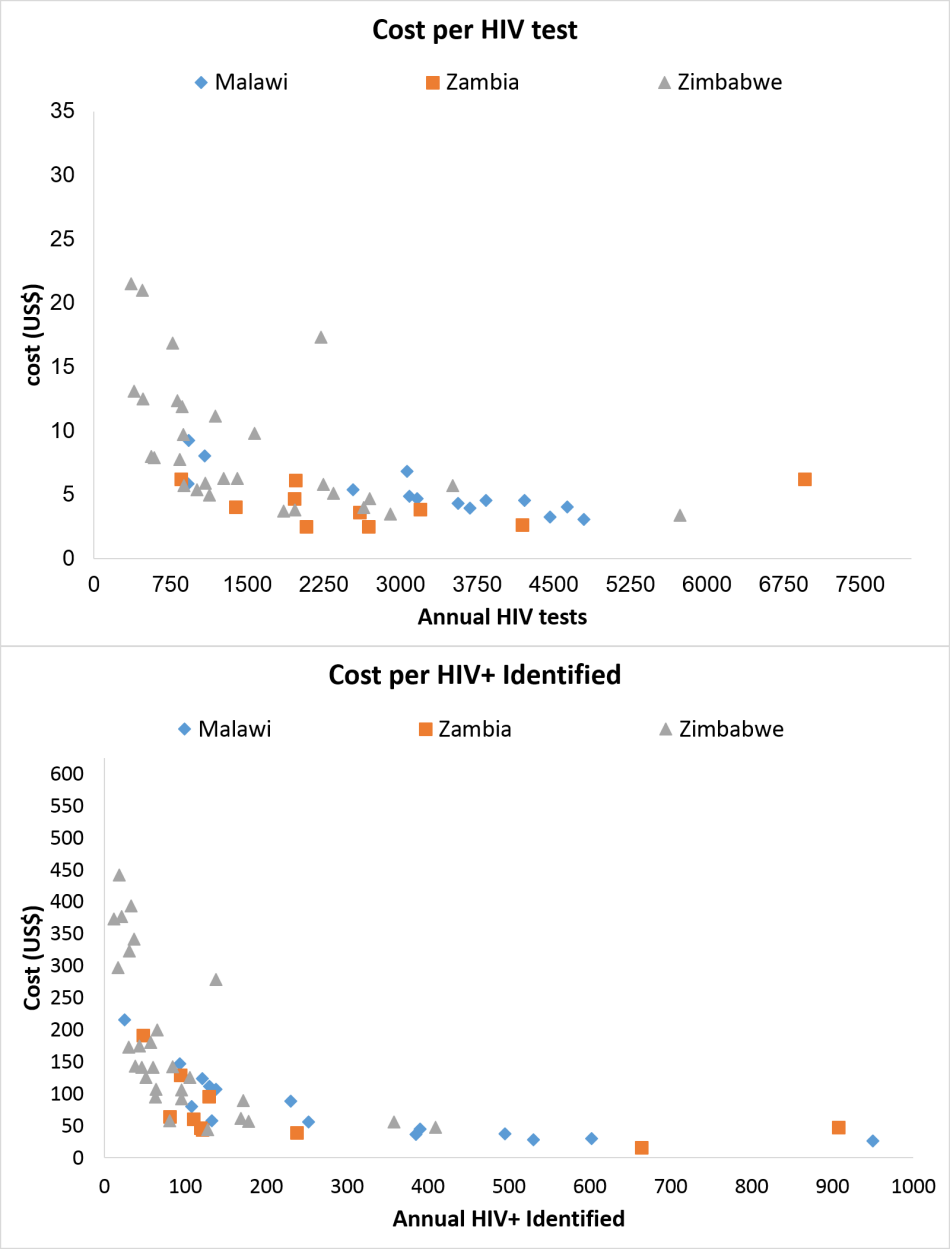
Economies of scale.

To identify the presence of economies of scale, Fig 3 shows the cost per individual tested and cost per HIV-positive individual identified by the annual number of HIV testing episodes performed at the health facility and the annual number of HIV-positive individuals identified at each of the health facilities, respectively. The cost per individual tested for HIV was lower at health facilities that were testing more individuals. Likewise, the cost per HIV-positive individual identified was lower at health facilities that were identifying more HIV-positive individuals.

### Sensitivity analysis

When varied the prices of HIV test kits from the observed prices for each country (base prices) to the observed minimum price (US$1.00 for Determine in Malawi and US$0.71 in Zimbabwe, both the mean cost per individual tested for HIV and mean cost per HIV-positive individual identified changed by 13% for Malawi, 11% for Zambia and 18% for Zimbabwe. When test kit prices were set at the observed maximum prices (US$1.10 for Determine and US$1.60 for UniGold in Zambia), the mean cost per individual tested for HIV changed by 11% for Malawi, 9% for Zambia and 13% for Zimbabwe. The mean cost per HIV-positive individual identified changed increased by the same magnitude for each country.

When we set personnel costs were set at 20% lower than actually observed, both the mean cost per individual tested for HIV and mean cost per HIV-positive individual identified reduced by 13% for Malawi, 11% for Zambia and 18% for Zimbabwe. When personnel costs were 20% higher than that observed, the mean cost per individual tested for HIV increased by 11% for Malawi, 9% for Zambia and 13% for Zimbabwe. The mean cost per HIV-positive individual identified increased by 10% for Malawi, 9% for Zambia and 13% for Zimbabwe. Only Zimbabwe included hospitals in the costing. When these were excluded, mean cost per individual tested for HIV ranged from US$8.79 to US$7.65, and mean cost per HIV-positive individual identified dropped from US$178.92 to US$150.40. Table 4 shows details of outcomes from sensitivity analysis.

**Table 4:** Sensitivity analysis results.

Parameter	Malawi (US$)		Zambia (US$)		Zimbabwe (US$)	
	Per HIV test	Per HIV+	Per HIV test	Per HIV+	Per HIV test	Per HIV+
**Base case**	5.05	79.58	4.24	73.63	8.79	178.92
***HIV Test kit Prices***						
Observed low prices (Determine = US$0.87; UniGold = US$0.71)	5.02	75.93	3.88	67.44	8.70	176.91
Observed Higher prices (Determine = US$1.10; UniGold = US$1.60)	5.22	82.05	4.24	73.63	8.93	181.88
*Personnel costs*						
20% reduction	4.45	70.27	3.83	66.26	7.45	152.68
20% increase	5.64	88.90	4.65	80.99	10.13	205.25

## Discussion

Health facility-based HIV testing remains the most common approach for individuals to learn their HIV status. Ensuring that 90% of all people living with HIV in sub-Saharan Africa know their HIV status by 2020 may require further scale-up of facility-based HTS. We found that the costs of delivering these HTS services in three southern African countries could be as low as US$3 per individual tested, especially in health facilities that were seeing a larger number of individuals.

**Fig 4:**
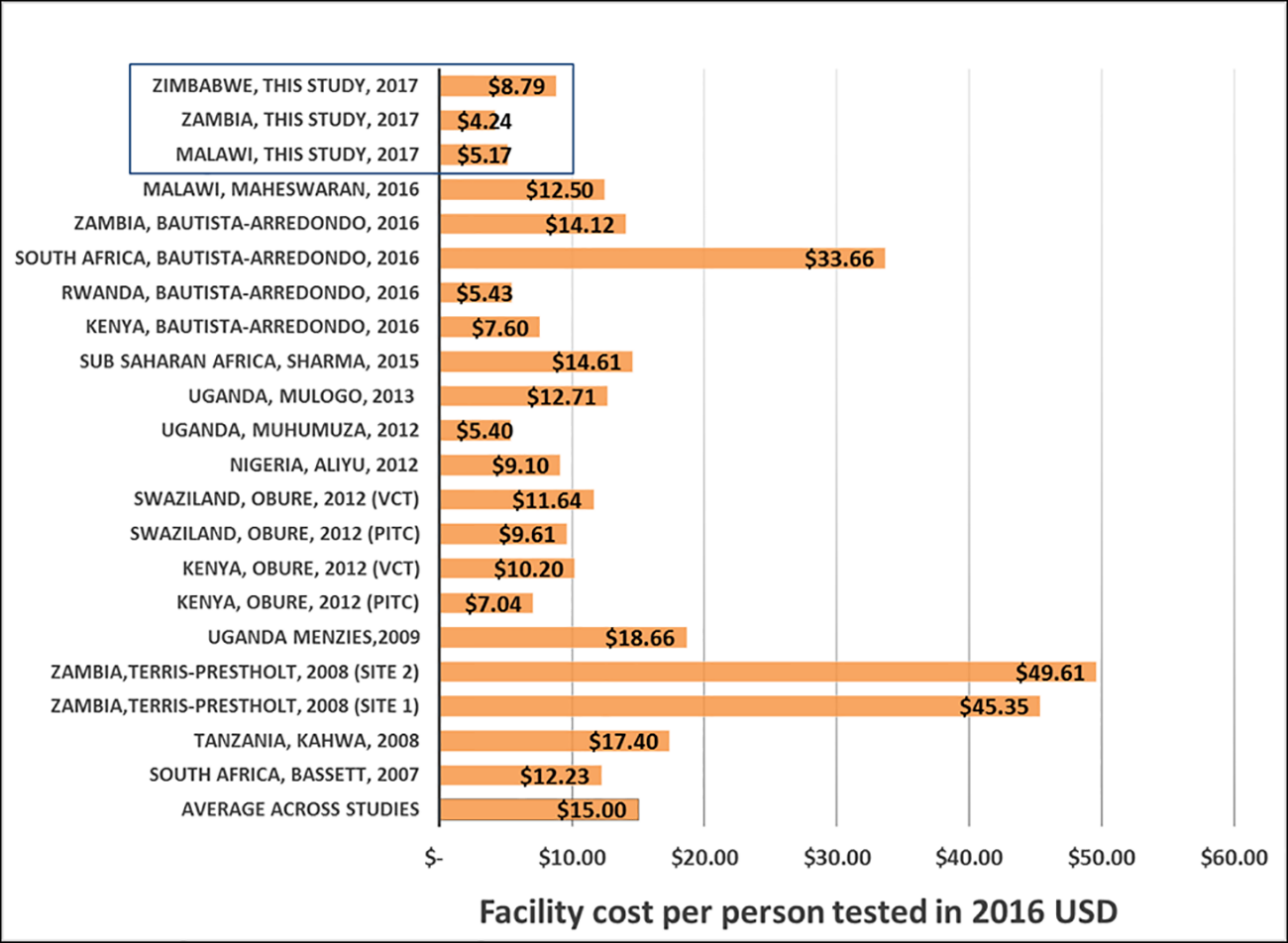
Comparison of cost per person tested for HIV in health facility in sub-Saharan Africa.

The mean provider costs of facility-based HTS were similar in Malawi and Zambia and higher in Zimbabwe, ranging from US$4.24 to US$8.79 per person tested. Our findings are fairly consistent with previous studies that estimated costs to test for and identify individuals with HIV at health facilities in the region (Fig 4) [29–39]. A facility-based costing study conducted in Malawi in 2014, with capital, overhead, staff salaries, consumables and equipment costs reported in 2014 prices, showed a higher cost of US$12.50 per person tested when adjusted to 2016 prices [40]. Notably, this estimate included costs of staff training, and service monitoring and evaluation, which was not observed in our study.

Previous studies in Zambia and South Africa, conducted between 2011 and 2012 with costs reported in 2013, estimated costs of US$14.12 and US$33.66 per person tested (in 2016 prices), respectively. Staff salaries were the main cost driver in South Africa [31]. The average economic costs were also estimated in 2009 for Kenya and Swaziland, with costs per person tested ranging from US$10.20 to US$11.64 for voluntary counselling and testing (VCT), and US$7.04 to US$9.61 (in 2016 prices) for provider initiated testing and counselling (PITC) [37]. These recent studies show large decreases as compared to cost estimates from the early years of HTS introduction (2001), costs reported in 2007, of which US$49.61 and US$45.35 (in 2016 prices) are reported costs per person tested [39]. It is important to note that, during early years of HTS introduction, HTS were delivered at high costs. HTS delivery was also surrounded by a lot of challenges (e.g. stigma, lack of confidentiality, fewer testing facilities) which required a lot of effort to create user demand [41–43]. Common across facility costing studies of HTS are the large contribution of human resources, training, test kits and consumables as drivers of costs.

We found considerable variation in cost estimates within and between countries and over time as the approach to and intensity of HTS evolved. Unit costs were especially low in larger health facilities that were seeing more individuals. These facilities often also provided a broader range of services. This suggests potential economies of scale, where inputs are more efficiently used due to fixed costs being spread across more outputs, and/or economies of scope, where fixed costs are spread across more services, both leading to lower unit costs. We did not find a strong relationship with the number of HIV counsellors working at the health facility and the number of individuals undergoing HIV testing. It is possible health facilities with greater numbers of HIV counselors are seeing fewer individuals for HIV testing during the time period of this study because past HIV testing was high and therefore fewer individuals in the community are unaware of their current HIV status. Conversely it is also possible that the demand for HIV testing amongst those served by these better staffed facilities, or the size of the facilities’ catchment population are low. However, the findings suggests that existing HTS in health facilities could be seeing more individuals for HIV testing without needing additional resources except the consumables needed to perform the HIV test. We found that the monthly number of HTS episodes at health facilities in Malawi gradually increased over the study period. This may reflect the recent introduction of test and treat, where HIV treatment is initiated immediately upon an HIV-positive test result [18]. Conversely, we found major fluctuation in the monthly number of HTS episodes at health facilities in Zimbabwe. This could be due to supply issues, e.g. HIV test kit stock outs. Alternatively, demand side variation, for example anecdotal evidence suggests peaks in rural HTS around the Christmas period and subject to weather conditions, that may universally affect people presenting for HTS.

Observed cost variation across countries and facilities presents a room for HTS innovations as well as an opportunity to assess the additional resources and approaches needed to achieve the UNAIDS 90-90-90 targets. For example, engaging communities through outreach programmes may complement facility-based HIV testing in settings with low demand [44]. Personnel costs accounted for a significant component of the total provider costs of facility-based HTS. There have been suggestions that the counselling process could be optimised [45], enabling counsellors to see more individuals or facilities to be staffed by fewer personnel. Alternatively, providing HIVST kits to health facility attendees, allowing them to perform and interpret their own test result, potentially in the privacy of their own homes or within private areas within facilities and discuss their results with healthcare providers. This approach could also reduce personnel needs at facilities or allow busy health facilities to meet HTS demand. HIVST has the additional benefit of high acceptability especially amongst men [46]. However, recognition of other potential bottle necks should be considered weighing the benefits of introducing new technological innovations because low output may also be caused by supply challenges such as stock-outs, which new test technology may or may not alleviate.

The cost per HIV-positive individual identified in our study ranged from as low as US$17 to as high as US$442. HIV testing and anti-retroviral treatment (ART) has been available in these three countries for over a decade, with recent estimates suggesting more than half of people living with HIV (PLHV) in the region are receiving treatment [1]. As there are fewer and fewer numbers of PLHV unaware of the infection, the cost per HIV-positive individual identified by HTS will continue to increase over time. In order to achieve the UNAIDS 90-90-90 targets this cost estimate should not inform decisions to fund or not fund HTS services, but may still provide useful insight into which HTS services are effective. It is important to note that we found approximately one in ten attendees of facility-based HTS in these three southern African countries to be HIV-positive. This confirms the fact that the three countries have made tremendous progress towards the 1st 90 of the USIAD 90-90-90 target [3, 4, 10], leading to having most of the people with known HIV status, and the remaining population comprising of ‘hard-to-reach people who may not want to test. Our study shows similar HIV reactivity rate (6–8%) across the three countries despite having quite different national HIV prevalence. This could be attributed to the fact that most of our facilities were rural with low population density and more importantly HIV prevention and treatment activities are widely provided in these communities with notable impact [3, 4, 10]. Health facilities continue to provide an important route for individuals to learn their HIV status.

A major limitation of our findings is the different financial reporting systems used in the three countries that made it challenging to standardise the allocation of central overhead costs. Another challenge in our data collection was that, as in other similar studies, we faced poor record keeping in the facilities; missing information and inconsistency in financial reporting across facilities. Additionally, by not including costs borne by patients and their carers for accessing testing, this does not give a true reflection of the economic burden of HIV testing. Measurement of patients’ costs can be essential for social planning as it gives insight into costs borne by individuals, households and society as a whole and can identify barriers to accessing HIV testing. However, an analysis of patient costs of accessing HTS in the same setting is underway. Thus, future research should consider direct and indirect costs of treatment from, at least, the provider and patient perspective as well as the long-term disability due to illness. This perspective can complement the provider’s perspective taken in this study.

Facility-based HIV testing services remains an effective approach to identifying undiagnosed HIV-positive individuals and can be an affordable approach to reaching the first 90. There are potential opportunities to improve their efficiency, which would need to be complemented by approaches to address demand side constraints to have a beneficial impact.

## Supporting information

S1 Fig Malawian HIV testing algorithm. (TIF)

S2 Fig Zambian HIV testing algorithm. (TIF)

S3 Fig Zimbabwe HIV testing algorithm for children above 18 months, adolescents and adults. (TIF)

S1 Table Cost allocation factors. (DOCX)

S2 Table Resource utilization of key HTS key supplies. (DOCX)

S3 Table Financial cost: Mean (min-max). (DOCX)
