# Supplementary figures and images for: Costs of facility-based HIV testing in Malawi, Zambia and Zimbabwe
Source: PLoS One. 2017 Oct 16;12(10):e0185740. doi: 10.1371/journal.pone.0185740 (PMC5642898; doi:10.1371/journal.pone.0185740)

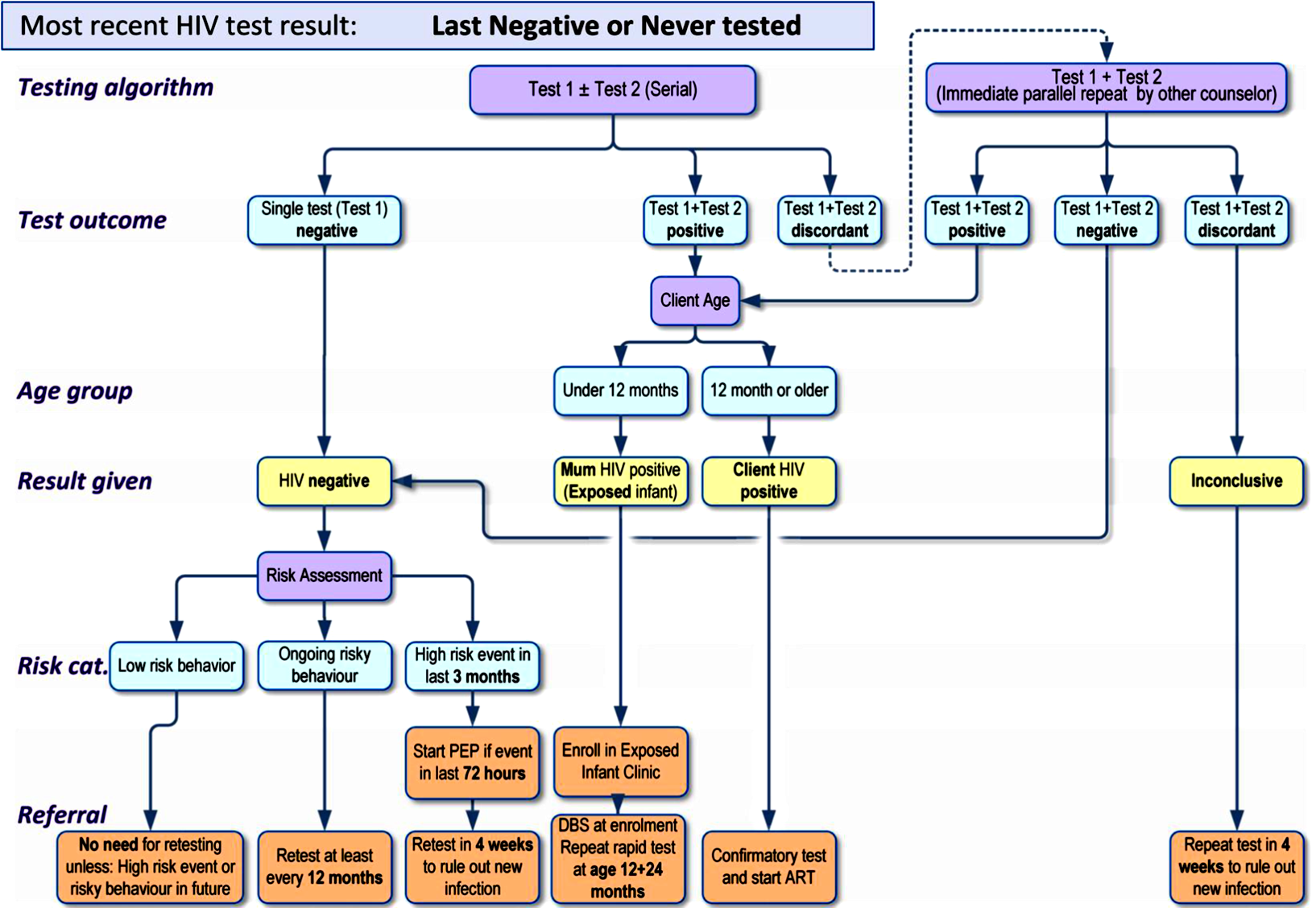

Supplement: S1 Fig — (TIF) [file pone.0185740.s001.tif]

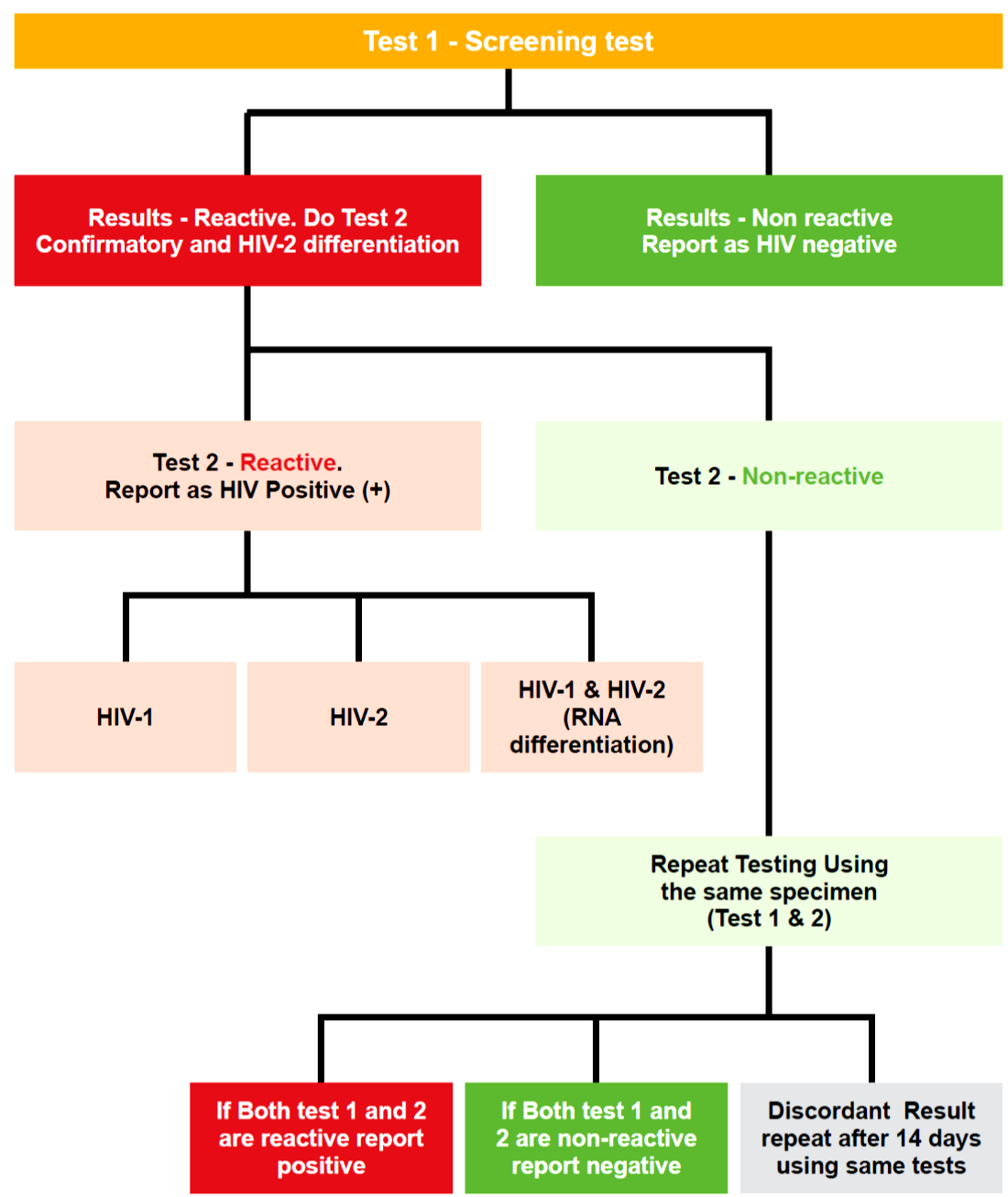

Supplement: S2 Fig — (TIF) [file pone.0185740.s002.tif]

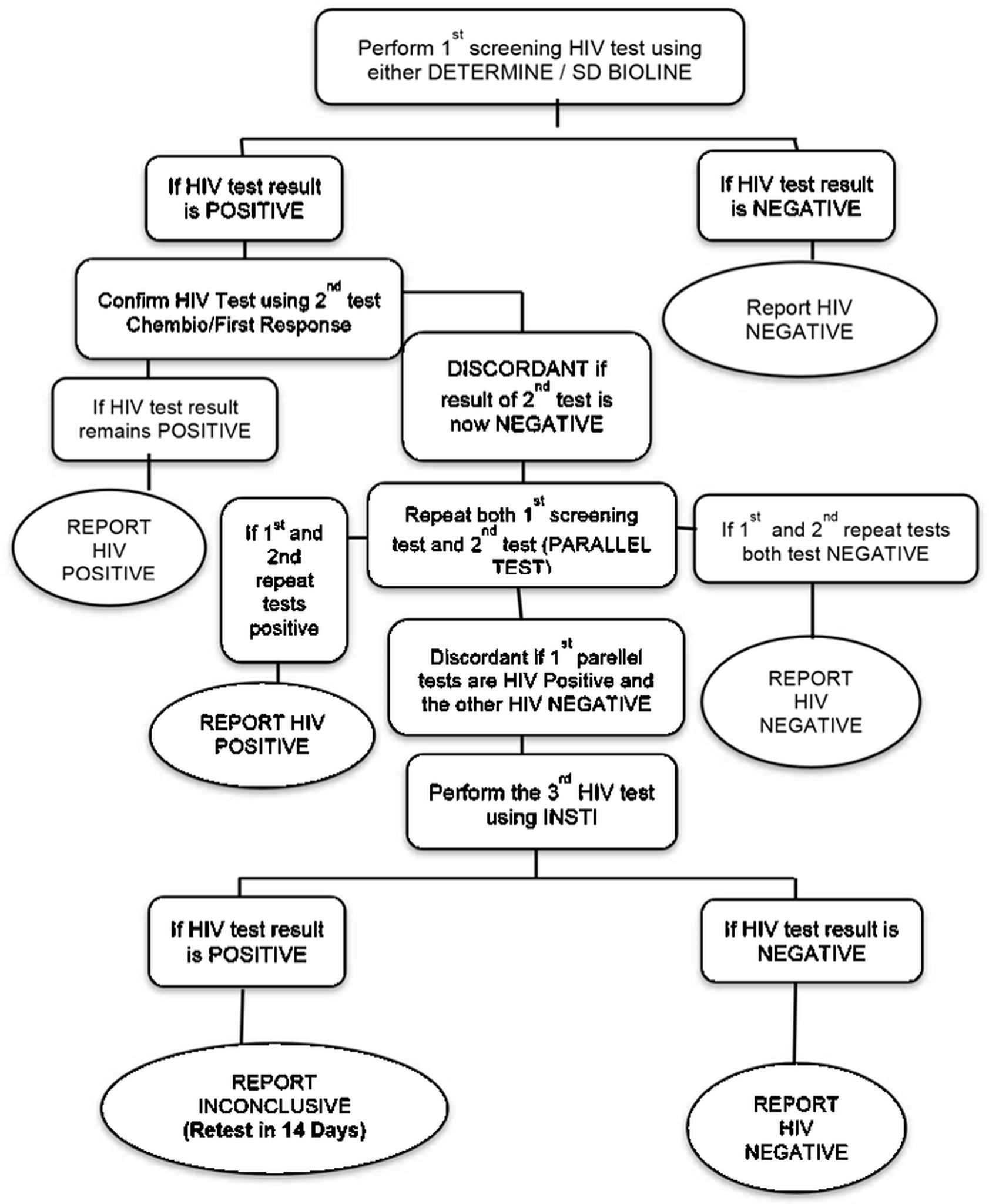

Supplement: S3 Fig — (TIF) [file pone.0185740.s003.tif]
