# Supplementary material for: Costs of facility-based HIV testing in Malawi, Zambia and Zimbabwe
Source: PLoS One. 2017 Oct 16;12(10):e0185740. doi: 10.1371/journal.pone.0185740 (PMC5642898; doi:10.1371/journal.pone.0185740)
Supplement: S1 Table — (DOCX) [file pone.0185740.s004.docx]

**S 1 Table. Cost allocation factors**

| Cost item | Zambia | Malawi | Zimbabwe |
| --- | --- | --- | --- |
| Health systems management (Above-facility administration, supervision & mentorship) | The proportion of facility catchment population relative to district catchment population then HTS proportion relative to total facility patient visits. | Proportion of HTS staff relative to total facility staff. | Based on number of supervisory day visits per year dedicated to each health facility i.e. once per quarter |
| Administrative Staff & office supply | Proportion of HTS staff relative to total facility staff. | Proportion of HTS staff relative to total facility staff. WHO-CHOICE annual facility staff stationery needs | Proportion of HTS staff relative to total facility staff. |
| Direct personnel | Full-time equivalent. | | |
| Shared direct costs (general supplies) | The proportion of HTS visit relative to total facility visits. | | |
| Waste management | Proportion HTS surface area relative to total Facility surface area. | | |
| Medical supplies and testing commodities | Directly allocated: quantified and valued by multiplying quantities and related unit costs | | |
| Supply chain costs | Secondary data[46] | | |
| * Health systems management costs were available by; district in Zambia and by facility in Malawi and Zimbabwe | | | |
