# Supplementary material for: Costs of facility-based HIV testing in Malawi, Zambia and Zimbabwe
Source: PLoS One. 2017 Oct 16;12(10):e0185740. doi: 10.1371/journal.pone.0185740 (PMC5642898; doi:10.1371/journal.pone.0185740)
Supplement: S2 Table — (DOCX) [file pone.0185740.s005.docx]

**S 2 Table. Resource utilization of Key HTS Key supplies**

| **Resource item*** | **Unit of measure** | **Unit costs** | **Average Quantity** |
| --- | --- | --- | --- |
| **Malawi** |  |  |  |
| *Personnel* | *staff cadre* | *Monthly Salary (US$)* | *Average annual number* |
| Lay counselor | volunteers | - | NIL |
| Psycho-social counselor | Paid counselor | 299.71 | 2 |
| *Consumables* | *unit of measure* | *Prices (US$)* | Average annual Quantity |
| Aprons | Each | 3.59 | 29 |
| Cotton wool | Roll of 500g | 4.15 | 11 |
| Determine | Each | 1.00 | 3,404 |
| Gloves | pack of 50 | 4.15 | 81 |
| Methylated Spirit | 1 litre | 4.02 | 9 |
| Registers | Each | 90.80 | 2 |
| Sharp boxes | Each | 2.00 | 18 |
| Unigold® | Each | 1.00 | 304 |
| **Zambia** |  |  |  |
| *Personnel* | *staff cadre* | *Monthly Salary (US$)* | *Average annual number* |
| Lay counselors | volunteers | 37.40 | 4 |
| Psycho-social counselor | Paid counselor | 259.31 | 1 |
| *Consumables* | *unit of measure* | *Prices (US$)* | Average annual Quantity |
| *Sharp boxes* | each | 16.80 | 8.00 |
| Determine test kit | pack | 901.20 | 29.44 |
| Unigold® | pack | 113.45 | 11.94 |
| Gloves | pack | 38.00 | 27.78 |
| Methylated Spirit | 2.5 litres | 6.97 | 4.24 |
| cotton Wool | roll of 500g | 7.40 | 4.44 |
| Medicated Soap | bar of 100g | 1.92 | 6.44 |
| Daily activity reports (printed material) | each | 8.48 | 4.00 |
| VCT Register | each | 24.93 | 0.25 |
| **Zimbabwe** |  |  |  |
| *Personnel* | *staff cadre* | *Monthly Salary (US$)* | *Average annual number* |
| Lay counselor | volunteers | - | NIL |
| Psycho-social counselor | Paid counselor | 384.00 | 5 |
| *Consumables* | *unit of measure* | *Prices (US$)* | *Average annual Quantity* |
| Determine Test Kits | Each | 1.07 | 1,606.65 |
| 1st Response Test kits (Bioline) | Each | 0.73 | 150.37 |
| Latex Gloves | Each | 0.09 | 1,544.59 |
| Cotton wool | Roll of 500g | 2.21 | 3.42 |
| Methylated Spirit | container of 750ml | 2.40 | 9.82 |
| HTC Register | Each | 9.50 | 2.97 |
| **Overhead/shared costs are not documented here because of variation in data collection methodology across countries* | | | |
