# Supplementary material for: Costs of facility-based HIV testing in Malawi, Zambia and Zimbabwe
Source: PLoS One. 2017 Oct 16;12(10):e0185740. doi: 10.1371/journal.pone.0185740 (PMC5642898; doi:10.1371/journal.pone.0185740)
Supplement: S3 Table — (DOCX) [file pone.0185740.s006.docx]

S 3 Table. Financial cost: mean (min-max)

| **Cost item** | **Malawi (US$)** | | | **Zambia (US$)** | | | **Zimbabwe (US$)** | | |
| --- | --- | --- | --- | --- | --- | --- | --- | --- | --- |
|  | **Total** | **Cost per test** | **Cost per HIV+** | **Total** | **Cost per test** | **Cost per HIV+** | **Total** | **Cost per test** | **Cost per HIV+** |
| **Capital costs** |  |  |  |  |  |  |  |  |  |
| *Buildings and Storage* | 236 | 0.08 | 1.23 | 82 | 0.04 | 0.6 | - | - | - |
|  | (31-704) | (0.01-0.18) | (0.20-4.88) | (36-156) | (0.01-0.13) | (0.10-1.15) |  |  |  |
| *Equipment* | 151 | 0.06 | 1.29 | 148 | 0.09 | 1.33 | 98 | 0.1 | 2.15 |
|  | (53-264) | (0.01-0.24) | (0.12-8.67) | (35-368) | (0.1-0.43) | (0.05-3.07) | (34-275) | (0.01-0.42) | (0.14-10.11) |
| *Vehicles* | - | - | - | 78 | 0.05 | 0.59 | 20 | 0.01 | 0.06 |
|  |  |  |  | (18-213) | (0.01-0.23) | (0.03-1.59) | (0.00-580) | (0.00-0.17) | (0.00-1.62) |
| *Other:* | - | - | - | 40 | 0.02 | 0.36 | - | - | - |
|  |  |  |  | (27-56) | (0.00-0.05) | (0.04-1.13) |  |  |  |
| ***Total capital*** | ***387*** | ***0.15*** | ***2.52*** | ***347*** | ***0.2*** | ***2.87*** | ***118*** | ***0.1*** | ***2.2*** |
|  | ***(85-967)*** | ***(0.02-0.37)*** | ***(0.44-13.55)*** | ***(117-793)*** | ***(0.04-0.84)*** | ***(0.24-5.92)*** | ***(34-855)*** | ***(0.01-0.42)*** | ***(0.14-10.11)*** |
| **Recurrent costs** |  |  |  |  |  |  |  |  |  |
| *Personnel* | 8375 | 2.89 | 46.49 | 5102 | 1.33 | 23.9 | 7670 | 6.69 | 131 |
|  | (2893-13828) | (1.32-5.41) | (13.05-115.72) | (457-30421) | (0.17-4.437) | (3.21-87.71) | (3141-34398) | (1.85-118.88) | (26.36-313) |
| *Supplies- Test kits* | 4164 | 1.19 | 19.16 | 3421 | 1.22 | 21.34 | 1826 | 1.2 | 29 |
|  | (1040-10093) | (1.13-1.26) | (8.51-41.58) | (1128-8692) | (1.14-1.35) | (8.21-46.39) | (439-6747) | (1.12-1.29) | (9.39-84.61) |
| *Supplies* | 1231 | 0.45 | 7.79 | 462 | 0.21 | 3.32 | 441 | 0.38 | 7.82 |
|  | (783-1632) | (0.14-0.98) | (1.22-31.32) | (206-596) | (0.08-0.58) | (0.62-5..95) | (130-2032) | (0.09-2.9) | (1.61-31.27) |
| *Supply chain* | 111 | 0.04 | 0.7 | 307 | 0.11 | 1.91 | 203 | 0.14 | 3.22 |
|  | (70-147) | (0.01-0.10) | (0.11-2.82) | (101-779) | (0.10-00.12) | (0.76-4.16) | (63-676) | (0.03-0.34) | (0.41-9.26) |
| *Operation & maintenance* | 393 | 0.36 | 3.64 | 751 | 0.42 | 6.85 | 56 | 0.1 | 0.7 |
|  | (67-1325) | (0.06-1.22) | (0.62-12.27) | (210-1427) | (0.05-1.14) | (0.32-13..71 | (0-682) | (0.00-01.15) | (0.00-8.42) |
| *Recurrent training* | - | - | - | - | - | - | - | - | - |
| *Waste management* | 31 | 0.01 | 0.23 | 2 | - | 0.02 | 2 | 0 | 0.06 |
|  | (2-136) | (0.00-0.05) | (0.02-1.46) | (1-4) |  | (0.00-0.07) | (0-6) | (0.00-0.01) | (0.00-0.37) |
| ***Total recurrent*** | ***14304*** | ***4.85*** | ***76.25*** | ***10045*** | ***3.29*** | ***57.34*** | ***10198*** | ***8.46*** | ***171.88*** |
|  | ***(4981-24228)*** | ***(2.96-8.90)*** | ***(25.50-199.22)*** | ***(3395-40827)*** | ***(1.95-5.87)*** | ***(16.21-156.34)*** | ***(4162-38155)*** | ***(3.33-20.68)*** | ***(41.96-426.04)*** |
| **Total cost/unit cost** | **14691** | **4.99** | **78.78** | **10392** | **3.5** | **60.21** | **10316** | **8.56** | **174.09** |
|  | **(5319-25042)** | **(3.02-9.14)** | **(26.36-212.77)** | **(4109-41076)** | **(2.05-5.90)** | **(16.45-162.26)** | **(4224-38252)** | **(3.34-20.77)** | **(42.36-431.10)** |
